# Identification and Characterization of lncRNA and mRNA in Testes of Landrace and Hezuo Boars

**DOI:** 10.3390/ani11082263

**Published:** 2021-07-30

**Authors:** Bo Zhang, Zunqiang Yan, Pengfei Wang, Qiaoli Yang, Xiaoyu Huang, Haixia Shi, Yuran Tang, Yanan Ji, Juanli Zhang, Shuangbao Gun

**Affiliations:** 1 College of Animal Science and Technology, Gansu Agricultural University, Lanzhou 730070, China; zhangb1662@163.com (B.Z.); yanzunqiang@163.com (Z.Y.); wangpf815@163.com (P.W.); yangql0112@163.com (Q.Y.); huanghxy100@163.com (X.H.); shxgsau@163.com (H.S.); tangyuran520@163.com (Y.T.); ji2660707355@163.com (Y.J.); zhangjuanli456888@163.com (J.Z.); 2 Gansu Research Center for Swine Production Engineering and Technology, Lanzhou 730070, China

**Keywords:** Hezuo boar, testicular development, spermatogenesis, precocious puberty, RNA-Seq

## Abstract

**Simple Summary:**

Precocious puberty is an excellent reproductive trait in domestic animals, which can generate higher breeding benefits in livestock production. However, regulators associated with this sexual maturation process remain largely unknown. Chinese Hezuo (HZ) boars are known for their early sexual maturity. In this work, the characteristics of precocious puberty in HZ pigs were confirmed by histological analysis, and some important long noncoding RNA (lncRNA) and mRNA were identified in the testes of immature (30-day-old) and mature (120-day-old) HZ boars, which could play a key role in precocious puberty. These results will provide a theoretical basis for further research on the regulatory mechanism of precocious puberty, which is important for accelerating the breeding process of highly fertile animals.

**Abstract:**

Chinese HZ boars are typical plateau miniature boars characterized by precocious puberty, which is closely related to testicular development and spermatogenesis. Accumulating evidence indicates that lncRNA is involved in the testicular development and regulation of spermatogenesis. However, little is known about the lncRNA precocious regulation in testicular development and spermatogenesis on early sexual maturity of HZ boars. Thus, we investigated the expression and characterization of lncRNA and mRNA in 30-day-old and 120-day-old HZ boar testes using transcriptome to explore precocious puberty. Landrace (LC) boar was treated as the control. Histological analyses indicated that HZ boar underwent puberty development at an earlier stage than LC boar and had achieved sexual maturity at 120 days old. RNA-Seq yielded a total of 187 lncRNAs and 984 mRNAs; these molecules were identified as possible candidates for precocious puberty. GO terms and KEGG pathways enrichment analyses revealed that the differentially expressed lncRNA and their targeted genes were involved in metabolic pathways regulating testis development and spermatogenesis, such as the PI3K-Akt, TGF-beta and Wnt pathways. Further screening, some lncRNA (such as *LOC102166140*, *LOC110259451,* and *MSTRG.15011.2*), and mRNA (such as *PDCL2*, *HSD17B4*, *SHCBP1L*, *CYP21A2,* and *SPATA3*) were found to be possibly associated with precocious puberty, which would add to our understanding of the molecular regulatory mechanisms of precocious puberty. This study provided valuable information for further study of the role of lncRNA and mRNA in the process of precocious puberty.

## 1. Introduction

Precocious puberty is an excellent reproductive trait in domestic animals and can improve breeding efficiency, but it is generally manifested as a pathological condition in humans [1]. Recently, many scholars have researched this excellent trait in domestic animals. De Camargo et al. found that the mutation of exon T/A (chr29:12,999) of *JY-1* gene in Nellore female cattle was significantly associated with sexual precocity [2]. Feng and Su et al. identified some candidate genes (including *KISS-1*, *GPR54*, *GNRH1*, *CACNA1C*) related to precocity in Jining gray goats. Additionally, alleles B and D of *GPR54* gene were significantly associated with this trait [3,4]. Kang et al. revealed that *CCT6A* plays an important role in the process of sexual prematurity in Jining Bairi chicken [5]. Ding et al. observed that various types of germ cells appeared in the testicular spermatogenic tubules of 75-day-old Meishan boars but not in 75-day-old Duroc boars, indicating that Meishan boar is characterized by early sexual maturity. Moreover, it was inferred that *NTF3* might be a candidate gene for precocious puberty in Meishan pigs [6]. The above research indicates that the sexual precocity candidate genes play important roles in the process of the early maturation of livestock and poultry. These genes might be used as molecular markers for the selection and breeding of precocity breeds. Therefore, it is valuable to study the excellent trait of sexual precocity in livestock and poultry for production applications.

LncRNA is most commonly defined as the transcripts by RNA polymerase II of more than 200 nucleotides [7]. In past decades, lncRNA was considered junk RNA and transcriptional noise along with other noncoding RNA [8]. However, accumulating evidence indicates that lncRNA participates in various important biological phenomena, such as organ development [9], control of nuclear architecture and translation [10], X inactivation [11], regulation of epigenetic modifications [12], imprinting genomic loci [13], and some disease [14]. LncRNA also plays an important role in testicular development and male germ cell proliferation. The testis is an important organ to ensure the coordination of spermatogenesis and androgen secretion [15]. Numerous lncRNA have been identified at specific stages of testicular and spermatogenesis in mouse [16], rat [17], human [18], and domestic animals (such as pig, cattle, and sheep) [19,20,21]. Most of these screened lncRNA target protein-coding genes through some important signaling pathways (such as Wnt, AMPK, and estrogen pathways) to regulate testicular development and spermatogenesis, which influence the process of sexual maturation. However, the detailed molecular regulatory mechanisms of these lncRNA remain largely unclear so far, especially for precocious puberty. Precocious puberty is one of the most important characteristics of the indigenous pig breed. Hezuo (HZ) pig is a typical plateau-type pig, which is mainly distributed in Xiahe, Zhuoni, and Diebu of Gannan Tibetan Autonomous Prefecture. The most prominent characteristics are small size, strong resistance to disease, and early sexual maturation. Compared with foreign pig breeds (such as Landrace and Yorkshire), HZ pigs have earlier sexual maturity and HZ boars have mature sperm in their testes at 120 days [22]. Thus, we sought 12 RNA-Seq libraries constructed and sequenced by Illumina-HiSeq^TM^ 4000 technology to identify lncRNA and mRNA from testicular tissues of 30-day-old (immature) and 120-day-old (mature) HZ boars, with Landrace (LC) boars as control. Additionally, we attempted to provide the first insight into the molecular regulatory mechanisms of sexual precocity in HZ boars.

## 2. Materials and Methods

### 2.1. Ethics Statement

This study was approved by the Committee for Animal Ethics of the College of Animal Science and Technology, Gansu Agricultural University (approval number 2006-398). All experimental protocols were conducted in accordance with the approved guidelines.

### 2.2. Animals and Testis Sample Collection

HZ boars and LC boars were provided by Gansu Sunxiang breeding Co., Ltd. (Zhuoni, Gansu, China). Each of three 30-day-old and 120-day-old HZ and LC boars were castrated to obtain testes samples. HZ and LC boars have the same genetic background, respectively. They were reared under the same natural conditions. All samples were immediately frozen in liquid nitrogen and stored at −80 °C until RNA extraction. A proportion of samples were fixed in Bouin’s solution for histological comparison.

### 2.3. Hematoxylin and Eosin Staining

Testes samples were fixed in Bouin’s solution for 72 h and then were processed for paraffin sections of 4–6 µm thickness. Testes sections were cut and subjected to hematoxylin and eosin (HE), then observed for comparative histological morphology.

### 2.4. RNA Extraction and Qualification

The total RNA of each sample was isolated using Trizol reagent kit (Invitrogen, Carlsbad, CA, USA). RNA quality was assessed on an Agilent 2100 Bioanalyzer (Agilent Technologies, Palo Alto, CA, USA) and checked using RNase-free agarose gel electrophoresis, and the samples with RNA integrity number scores higher than seven can be used in this study.

### 2.5. Library Preparation and Sequencing

Approximately 3 µg total RNA per testis sample was used for preparing RNA-Seq libraries. Ribosomal RNAs (rRNAs) were removed using a Ribo-Zero Gold rRNA Removal Kit (Illumina, San Diego, CA, USA) to retain mRNAs and ncRNAs. The enriched mRNAs and ncRNAs were fragmented into short fragments using a fragmentation buffer (New England Biolabs, Beverly, MA, USA) and reverse transcribed into cDNA with random primers. Second-strand cDNA were synthesized by DNA polymerase I, RNase H, dNTP (dUTP instead of dTTP), and buffer. Next, the cDNA fragments were purified with QiaQuick PCR extraction kit (Qiagen, Venlo, The Netherlands), end repaired, poly(A) added, and ligated to Illumina sequencing adapters. Then, UNG (Uracil-N-Glycosylase) was used to digest the second-strand cDNA. The digested products were size selected by agarose gel electrophoresis, PCR amplified, and sequenced using Illumina-HiSeq^TM^ 4000 to generate PE150 reads by Gene Denovo Biotechnology Co. (Guangzhou, China). Raw data generated from this study were deposited in NCBI GEO (GSE171756).

### 2.6. Transcriptome Assemble

Raw reads were first subjected to quality filtering by fastp (V0.18.0) [23], which removed the reads containing less than 10% of unknown nucleotides (N), and low-quality reads (>50% of the bases with *q* value ≤ 20) to generate clean reads. All the downstream analyses were based on the high-quality clean data. Bowtie2 (V2.2.8) [24] was used for mapping reads to the ribosome RNA (rRNA) database. The rRNA mapped reads were removed, then the remaining reads were mapped to the reference genome (*Sus scrofa* 11.1, NCBI, https://www.ncbi.nlm.nih.gov, accessed on 8 April 2021) using HISAT2 (V2.1.0) [25] tool and other parameters set as a default. The mapped reads from each library were assembled with StringTie (V1.3.4) [26].

### 2.7. Gene Expression Quantification

The FPKM (fragment per kilobase of transcript per million mapped reads) was used to normalize the expression levels of lncRNA and mRNA. The FPKM eliminated the effect of sequencing depth, gene length, and sample difference on gene expression levels [27]. The significance of the differentially expressed lncRNAs and mRNAs were analyzed using an R package DESeq2 [28], and multiple comparisons were corrected for *p* value with the false discovery rate (FDR). A *q* value < 0.05 and absolute value of log_2_FoldChange >1 were used as the threshold to statistically define the significant difference.

### 2.8. Identification of Potential lncRNA Candidates

The following series of highly stringent filtering criteria were performed to identify lncRNA: (1) Transcripts overlapping with pig protein-coding genes were removed. (2) The length of the transcript was longer than 200 bp, and the exon number was more than two. (3) LncRNAs were classified into five classes (intergenic lncRNA, bidirectional lncRNA, intronic lncRNA, antisense lncRNA, and sense lncRNA.) according to their location relative to protein-coding genes. (4) We compared with annotation files to exclude known mRNA and other noncoding RNAs (such as rRNA, tRNA, snoRNA, snRNA). (5) Transcripts without coding potential, as predicted by CNCI [29], CPC2 [30], CPAT [31], and PFAM [32], were novel lncRNA.

### 2.9. Target Gene Prediction and Functional Enrichment Analysis

LncRNA can *cis*-regulate neighboring target genes and *trans*-regulate distant target genes. *Cis* activity in lncRNA is associated with interactions with neighboring target genes. We searched for protein-coding genes 10 kb up and downstream of each putative lncRNA [33]. *Trans* interactions are identified by the expression levels of lncRNA to each other; we calculated the expression correlation between lncRNA and coding genes using a custom script and identified the target genes using a Pearson correlation coefficient with absolute value >0.95 [34]. These target genes and the differentially expressed mRNAs were subjected to functional exploration according to the Gene Ontolog (GO) and Kyoto Encyclopaedia of Genes and Genomes (KEGG) enrichment analysis using DAVID online analytical tools to predict their functional roles, respectively [35]. The calculated *p* value went through FDR correction, taking the *q* value < 0.05 as a threshold. GO terms and KEGG pathways with a *q* value < 0.05 were considered to be significantly enriched.

### 2.10. LncRNA–mRNA Network Construction

To further explore the interactions between the differentially expressed lncRNAs and mRNAs of precocious puberty in HZ pigs, we constructed networks based on the targeting relationship between lncRNA and mRNA. Visualization of gene interactions was achieved through Cytoscape (V3.7.2) [36].

### 2.11. Validation of Differentially Expressed Genes by qRT-PCR

In order to validate the authenticity of RNA-seq data, a total of 16 differentially expressed genes (including 8 lncRNA and 8 mRNA) were selected for qRT-PCR analysis. Using the individual RNA samples, total RNA extracted originally for sequencing was converted to cDNA using a *Evo M-MLV* RT Kit with gDNA Clean for qPCR II (Accurate Biotechnology, Hunan, China). All experiments were performed in triplicate. LncRNA and mRNA primers are shown in Table S1. The qRT-PCR was performed in a LightCycler 480 II instrument (Roche, Basel, Switzerland) in reactions containing the 2×SYBR^®^ Green Pro Taq HS Premix II (Accurate Biotechnology, Hunan, China). The thermal cycler program included an initial denaturation at 95 °C for 3 min, followed by 40 cycles at 95 °C for 15 s; 57 °C for 15 s, and 72 °C for 20 s. All amplifications were followed by dissociation curve analysis of the amplified products. Gene expression was quantified relative to *β*-actin expression using the 2^−∆∆Ct^ method [37].

## 3. Results

### 3.1. Morphology of Testicular Tissues

**Figure 1:**
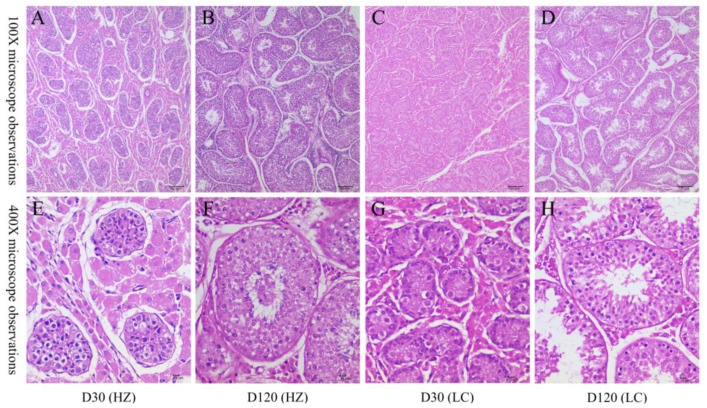
Histological evaluation of HZ and LC pig testicular tissues at 30 and 120 days old. The morphology of each sample was observed under light microscopy: (**A**–**D**) represent the testicular cross-sections of 30-day-old HZ, 120-day-old HZ, 30-day-old LC, and 120-day-old LC pig at 100x magnification, respectively; (**E**–**H**) represent the testicular cross-sections of 30-day-old HZ, 120-day-old HZ, 30-day-old LC, and 120-day-old LC pig at 400× magnification, respectively.

The morphological characteristics of testicular tissues were found to obviously change with age by sectioning and HE staining of testis tissues of 30-day-old and 120-day-old of HZ and LZ boars (Figure 1). With an increase in age, the diameter and the lumen area of convoluted seminiferous tubules increased obviously. Further, while the area of the visual field occupied by interstitial connective tissue was reduced, the number of spermatogenic cells and Sertoli cells increased. In addition, the diameter of the spermatogenic tubules was larger in the 30-day-old HZ boar compared with the testes of the LC boar. The type and number of spermatogenic cells were more abundant in the 120-day-old HZ boar compared with the testes of the LC boar, and mature spermatozoa were observed (Table S2). These results showed that the testicular development of HZ boars was earlier than that of LC pigs, and the time of sexual maturity was earlier than that of LC pigs.

### 3.2. Quality Analysis of RNA-Seq Data

A total of 12 cDNA libraries (Ha1, Ha2, Ha3, Hb1, Hb2, Hb3, La1, La2, La3, Lb1, Lb2, Lb3) were constructed for testis tissues from the HZ and LC boars at 30-day-old and 120-day-old, and each was sequenced using Illumina-HiSeqTM 4000. Ha, Hb, La, and Lb represent 30-day-old HZ boar, 120-day-old HZ boar, 30-day-old LC boar, and 120-day-old LC boar, respectively. An average of 100960468, 87829094, 93821582, and 93563089 raw reads were generated from 30-day-old HZ, 120-day-old HZ, 30-day-old LC, and 120-day-old LC boar testes, respectively. After discarding reads with poly-N >10%, poly-A, adapter, and other low-quality reads, an average of 100604464, 87609919, 93507526, and 93347563 clean reads were collected, respectively. The percentage of clean reads in each library ranged from 99.64 to 99.78%. The Bowtie2 was used for mapping reads to the ribosome RNA (rRNA) database, and the rRNA mapped reads were removed. These remaining reads were then aligned into the porcine reference genome (*Sus scrofa* 11.1) using the HISAT2 software. Approximately 95.58 to 97.15% of total clean reads were mapped to the porcine reference genome, 90.60 to 92.69% of the reads were uniquely mapped to specific regions of the porcine reference genome, and 3.88 to 5.23% of the sequences have multiple genome locations, respectively. These results indicated that the sequencing data of porcine testicular tissue are reliable and can be used for subsequent bioinformatics analysis (Table S3). The mapped sequences in each library were reconstructed and assembled using the Stringtie software, and a total of 15516 unique assembled transcripts were obtained. High-quality libraries are essential for lncRNA sequencing. Therefore, the data from each read’s relative position in the gene (5′-3′) were analyzed to ensure the quality of these 12 samples. Indeed, the vast majority of reads were evenly distributed throughout the gene, indicating good sample quality (Figure S1). Moreover, the ratio of reads corresponding to exon, intron, and intergenic regions was different, with exons accounting for the highest ratio, introns the next, and intergenic regions the lowest (Figure S2). These results indicated that RNA expression profiles were specific among different samples.

### 3.3. Identification and Characteristics of lncRNA and mRNA

**Figure 2:**
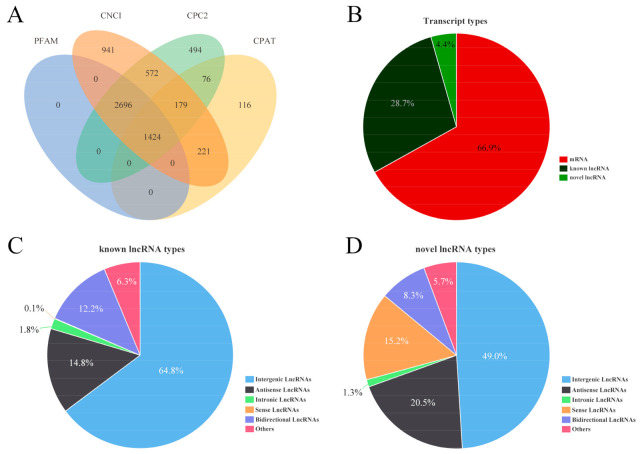
(**A**) Identification of 1424 putative lncRNAs without protein-coding potential evaluated by CNCI, PFAM, CPC2, and CPAT; (**B**) classification of transcripts; (**C**) known lncRNAs; (**D**) novel lncRNAs.

**Figure 3:**
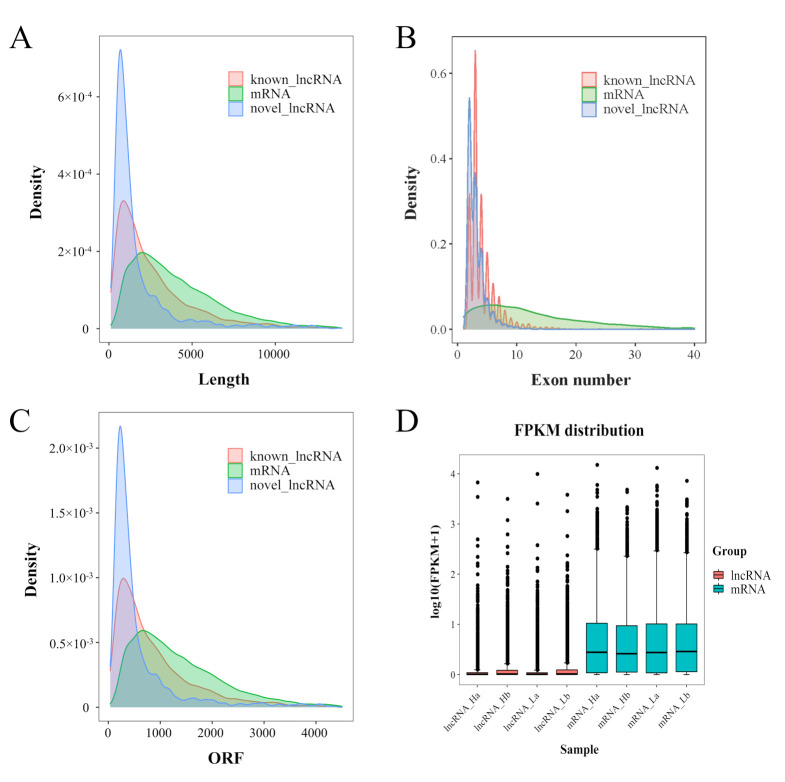
Genomic characteristics of lncRNA and mRNA in pig testes: (**A**) Length distribution of 9282 known lncRNAs (red), 21,674 mRNAs (green), and 1424 novel lncRNAs (blue); (**B**) exon number distribution of lncRNAs and mRNAs; (**C**) ORF length distribution of lncRNAs and mRNAs; (**D**) box plots showing the expression levels {log_10_(FPKM+1)} of lncRNA and mRNA in Ha, Hb, La, and Lb groups.

To identify the high-confidence lncRNAs, a stringent filtering pipeline was developed to discard some transcripts that did not have all the characteristics of lncRNA (Figure S3). Four bioinformatic tools (namely CNCI, CPC2, CPAT, and PFAM) were used to assess the protein-coding potential of novel transcripts. Then, the intersection of four non-protein-coding potential results was regarded as novel lncRNA. Through this strategy, a total of 10,706 lncRNAs (including 9282 known lncRNAs and 1424 novel lncRNAs) were identified in the testicular tissues in the 12 boars (Figure 2A). The 9282 known lncRNAs were classified into 6014 intergenic lncRNAs, 1375 antisense lncRNAs, 169 intronic lncRNAs, 7 sense lncRNAs, 1135 bidirectional lncRNAs, and 582 others lncRNAs, accounting for 64.8%, 14.8%, 1.8%, 0.1%, 12.2%, and 6.3%, respectively (Figure 2C). These known lncRNA transcripts corresponded to 1909 lncRNA genes. The 1424 novel lncRNAs were classified into 698 intergenic lncRNAs, 292 antisense lncRNAs, 18 intronic lncRNAs, 217 sense lncRNAs, 118 bidirectional lncRNAs, and 81 others, accounting for 49%, 20.5%, 1.3%, 15.2%, 8.3%, and 5.7%, respectively (Figure 2D). In addition, a total of 21,674 mRNAs were identified (Figure 2B). Subsequent analyses were based on these lncRNAs and mRNAs. We also characterized transcript length, the exon number, and open reading frame (ORF) length to explore differences of known and novel lncRNAs and mRNAs. Our results showed that the transcript length and ORF length of known and novel lncRNAs were significantly shorter than those of the mRNA transcript. Moreover, the number of exons was also less than that of mRNAs (Figure 3A–C).

### 3.4. Differential Expression Analyses of lncRNA and mRNA

**Figure 4:**
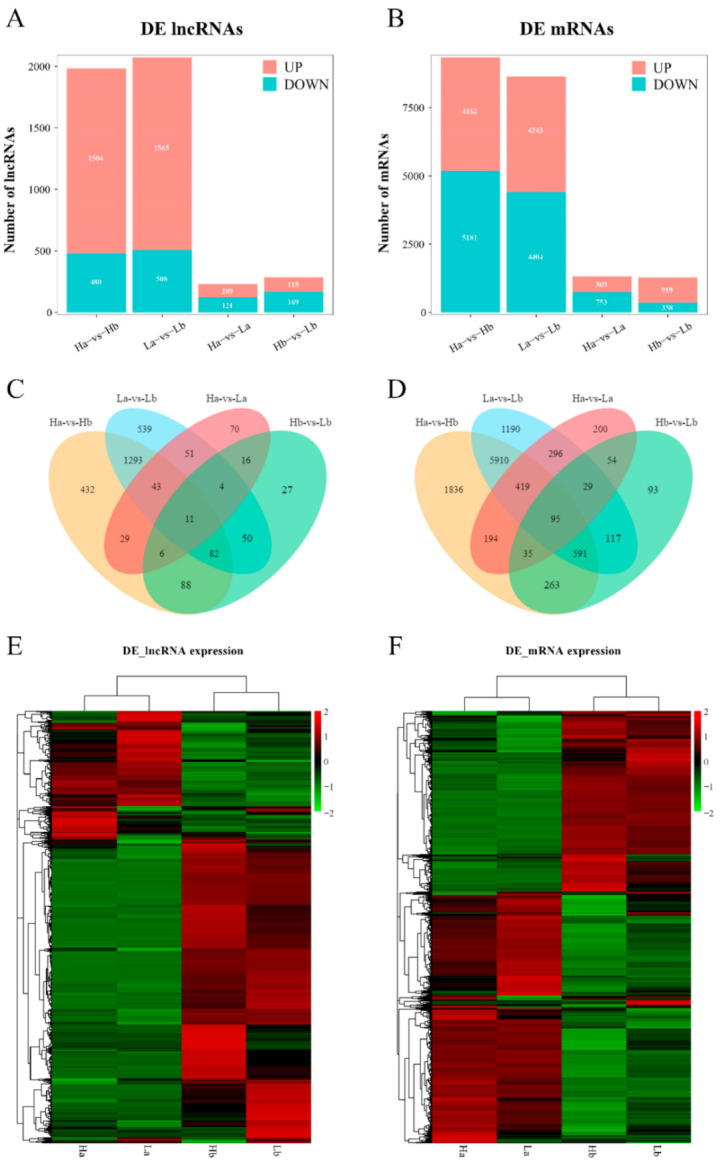
Gene expression number and profiling analyses of differentially expressed lncRNAs and mRNAs among Ha, Hb, La, and Lb groups: Statistics of differentially expressed lncRNAs (**A**) and mRNAs (**B**) among groups; Venn diagram of differentially expressed lncRNAs (**C**) and mRNAs (**D**) in four comparison groups; cluster analyses of differentially expressed lncRNAs (**E**) and mRNAs (**F**) of 12 pig testes by hierarchical heat map. Data are expressed as FPKM. Red and green represent higher expression levels and lower expression levels, respectively.

To assess the expression levels of lncRNA and mRNA, FPKM was calculated using StringTie to normalize the expression levels of lncRNA and mRNA. The expression level of lncRNA was much lower than that of mRNA (Figure 3D). Based on the expression of lncRNA and mRNA in each sample, we conducted principal component analysis (PCA) to confirm the reliability that the samples of each group are different from each other (Figure S4). DESeq2 software was applied to screen differentially expressed lncRNA and mRNA, the expression differences of lncRNA and mRNA were remarkable in the same breeds at different days than that in different breeds of the same age. With a *q* value < 0.05 and an absolute value of log_2_FoldChange >1, a total of 2741 DE lncRNAs and 11,322 DE mRNAs were screened in the testes of 30-day-old and 120-day-old HZ and LC boars, respectively. However, the difference was much smaller in different breeds of the same age. A total of 1984, 230, 284, and 2073 lncRNAs and 9343, 1322, 1277, and 8647 mRNAs were differentially expressed between Ha vs Hb, Ha vs La, Hb vs Lb, and La vs Lb groups (Figure 4A,B). These results indicated that the number of upregulated lncRNAs was more than the downregulated lncRNAs at different ages in the same breed. A Venn diagram was used to describe the overlaps between differentially expressed lncRNAs and mRNAs from pairwise comparisons. Among 2741 DE lncRNAs and 11,322 DE mRNAs, 11 and 95 co-existed in the within-breed and inter-breed comparisons, respectively (Figure 4C,D). In addition, the heat map showed that lncRNA and mRNA expression patterns between Ha, Hb, La, and Lb were distinguishable. A substantial difference exists between the Ha vs Hb and La vs Lb group, and the Ha vs La and Hb vs Lb showed a similar pattern. These results indicated that gene expression differences were more remarkable in testes at different ages than in different pig breeds (Figure 4E,F).

### 3.5. GO Terms and KEGG Pathways Analysis of Differentially Expressed mRNA

GO terms and KEGG pathways enrichment methods were conducted to assess differentially expressed mRNA function. GO annotation was categorized into biological processes (BP), cellular components (CC), and molecular function (MF). A total of 619, 514, 44, and 351 highly enriched GO terms were derived from Ha vs Hb, La vs Lb, Ha vs La, and Hb vs Lb groups, respectively (*q* value < 0.05, Table S4); The most enriched GO terms in four comparison groups included cellular developmental process (GO: 0048869), single–multicellular organism process (GO: 0044707), cell morphogenesis (GO: 0000902), calcium ion binding (GO: 0005509), transforming growth factor beta binding (GO: 0050431), and oxidoreductase activity (GO: 0016491). These differentially expressed mRNAs were involved in the development and change of the cell body and some other important functions. Obviously, we found some GO terms related to testicular development and spermatogenesis, such as male gamete generation (GO: 0048232), sexual reproduction (GO: 0019953), multicellular organism reproduction (GO: 0032504), spermatogenesis (GO: 0007283), and sperm flagellum (GO: 0036126). KEGG pathways analysis revealed that differentially expressed mRNAs were assigned to 107, 106, 4, and 22 pathways in Ha vs Hb, La vs Lb, Ha vs La, and Hb vs Lb groups, respectively (*q* value < 0.05, Table S5). These pathways included ECM–receptor interaction (ko04512), calcium (ko04020), PI3K-Akt (ko04151), MAPK (ko04010), Ras (ko04014), and Relaxin (ko04926). These results indicated that differentially expressed genes in the testes of HZ and LC boars were involved in testicular development and spermatogenesis through the above pathways.

### 3.6. GO Terms and KEGG Pathways Enrichment Analyses of Target Genes of Differentially Expressed lncRNA

To gain insight into the potential functions of differentially expressed lncRNAs in regulating precocious puberty associated with testicular development and spermatogenesis, GO and KEGG enrichment analyses were performed to investigate the roles of lncRNA in *cis*- and *trans*-regulation. We searched for protein-coding genes 10 kb up and downstream of all the identified lncRNAs as the *cis*-target gene. The results showed that 688 lncRNAs were near 603 mRNAs, corresponding to 795 lncRNA-coding gene pairs (Table S6). A total of 430 GO terms were significantly enriched (*p* value < 0.05, Table S7). The top five GO terms were cilium morphogenesis (GO: 0060271), nitrogen cycle metabolic process (GO: 0071941), microtubule organizing center (GO: 0005815), cellular response to xenobiotic stimulus (GO: 0071466), and S100 protein binding (GO: 0044548). A total of 17 pathways were significantly enriched (*p* value < 0.05, Table S8). These mainly included antigen processing and presentation(ko04612), TGF-beta(ko04350), carbon metabolism (ko01200), and biosynthesis of amino acids(ko01230)signaling pathways. Some target genes of these lncRNA were also found to enrich in some testicular development or spermatogenesis signaling pathways, such as PI3K-Akt (ssc04151) in Leydig cells [38] and Sertoli cells [39], MAPK (ssc04010) in germline stem cells [40], Sertoli cells [41], Leydig cells [42], and blood-testis barrier [43]. These findings suggested that lncRNA play an important role in pig testis development and spermatogenesis by acting on their neighboring protein-coding genes via *cis* pattern.

We also observed a correlated expression pattern of lncRNA and protein-coding genes in a *trans* manner. A total of 476,142 pairs were detected from 2346 lncRNAs and 9450 target genes using the absolute value of Pearson correlation >0.95 as the cut-off (Table S9). GO enrichment analysis results showed that 603 GO terms were significantly enriched (*q* value < 0.05, Table S10) and involved in protein binding (GO: 0005515), male gamete generation (GO: 0048232), sexual reproduction (GO: 0019953), sperm part (GO: 0097223), spermatogenesis (GO: 0007283), and multicellular organism reproduction (GO: 0032504). In addition, a total of 97 pathways were identified (*q* value < 0.05, Table S11); some pathways were related to testis development or spermatogenesis, such as the Ras (ssc04014) in spermatogonial stem cells [44], seminiferous epithelium cells [45], and testicular interstitial cells [46], AMPK (ssc04152) in Sertoli cells [47], and the TGF-beta (ssc04350) in testicular. These studies found that these lncRNA were involved in signaling pathways through target genes to affect testicular development, spermatogenesis, and maturation.

### 3.7. GO Terms and KEGG Pathways Enrichment Analysis of Genes Related to Precocious Puberty

**Figure 5:**
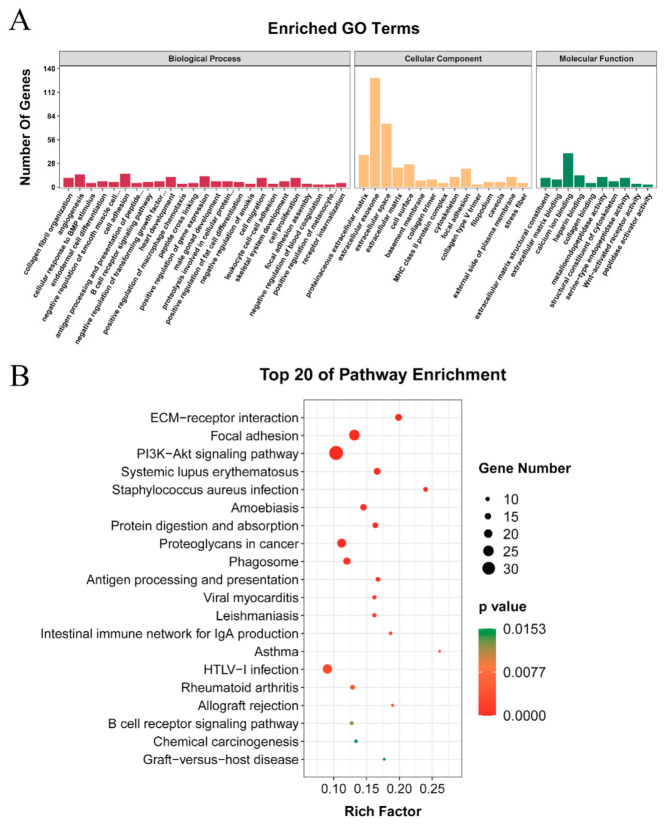
Functional enrichment analyses of the candidate genes: (**A**) GO functional annotation of the candidate genes related to precocious puberty. The X-axis indicates the detailed terms, and the Y-axis indicates the gene numbers; (**B**) KEGG pathways of candidate genes related to precocious puberty. The X-axis indicates the gene ratio, and the Y-axis indicates the name of KEGG pathway. The size of dots indicates the numbers of genes, and the color of dots indicates *p* value.

In order to find molecules, GO functions, and KEGG signaling pathways related to precocious puberty from massive data in HZ boars, we screened a collection of DE lncRNA and DE mRNA from Ha vs Hb and Hb vs Lb groups. These molecules (187 lncRNAs and 984 mRNAs) contained important information during testicular development in HZ boars and the differences of sexual maturity in HZ and LC boars, and important contributing molecules to precocious puberty in HZ pigs. (Table S12). A total of 157 GO terms and 49 signaling pathways were enriched. These GO terms were involved in collagen fibril organization (GO: 0030199), angiogenesis (GO: 0001525), cell adhesion (GO: 0007155), heart development (GO: 0007507), and male gonad development (GO: 0008584) (Figure 5A). The signal pathways include ECM–receptor interaction (ssc04512), PI3K-Akt (ssc04151), Wnt (ssc04310), TGF-beta (ssc04350) and Glycolysis/Gluconeogenesis (ssc00010) signaling pathways (Figure 5B). This implies that these molecules may play a key role in regulating precocious puberty through the above signaling pathways.

### 3.8. LncRNA–mRNA Interaction Network Analysis

**Figure 6:**
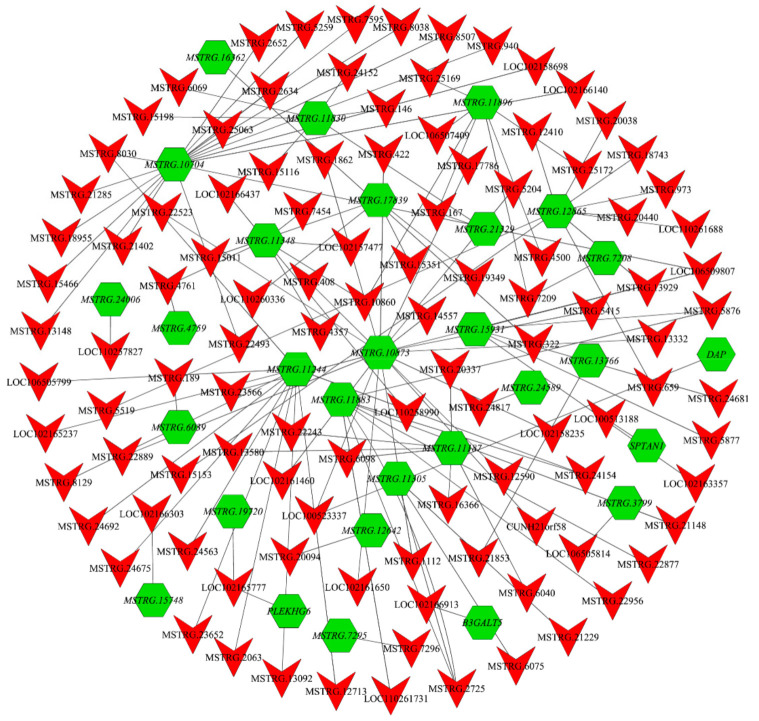
The network between DE lncRNAs and target DE mRNAs. Nodes represent lncRNA or mRNA, and edges represent the interaction between lncRNA and mRNA. Inverted triangles (red, regular) and hexagons (green, italic) represent lncRNA and mRNA, respectively.

Based on screened candidate molecules associated with precocious puberty, we predicted target genes (*cis* and *trans*) of the screened 187 lncRNAs and intersected them with the screened 984 mRNAs to finally obtain DE lncRNA and target DE mRNA. These differentially expressed molecules were used in the construction of the lncRNA–mRNA co-expression network (Figure 6). The whole co-expression network consisted of 145 network nodes and 122 connections among 119 DE lncRNAs and 29 DE mRNAs. Some mRNAs were centers of the network, such as *MSTRG.10873*, *MSTRG.11244,* and *MSTRG.10704*. Moreover, these results showed that one mRNA is associated with many lncRNAs, and one lncRNA is associated with many mRNAs, indicating that the interaction of lnRNA and mRNA participate in precocious puberty.

### 3.9. Validation of Expression Levels of lncRNA and mRNA Detected in RNA-Seq

**Figure 7:**
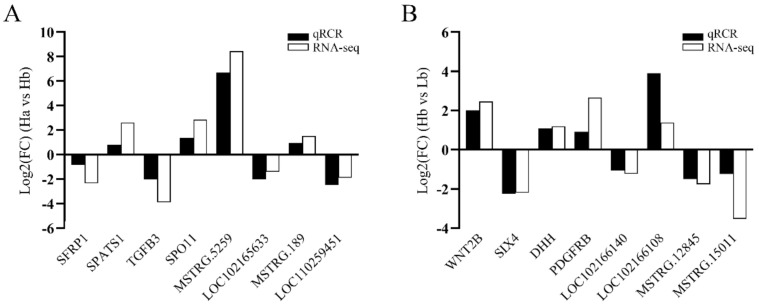
qRT-PCR verification of the differentially expressed genes: (**A**) The qRT-PCR verification of the differentially expressed genes in Ha vs Hb; (**B**) the qRT-PCR verification of the differentially expressed genes in Hb vs Lb.

To further validate the RNA-Seq result and detect expression level, we randomly selected 16 differentially expressed lncRNAs and mRNAs to conduct qRT-PCR detection, which included eight lncRNAs (*LOC102165633*, *LOC110259451*, *LOC102166140*, *LOC102166108*, *MSTRG.189*, *MSTRG.12845*, *MSTRG.15011*, *MSTRG.5259*) and eight mRNAs (*SFRP1*, *WNT2B*, *SIX4*, *TGFB3*, *DHH*, *SPATS1*, *SPO11*, *PDGFRB*). The expression of each lncRNA or mRNA was consistent with those obtained by sequencing (Figure 7), suggesting the high reliability and accuracy of RNA-Seq data.

## 4. Discussion

Improvements in pig reproduction performance are critical in livestock production, which can generate higher breeding benefits. Precocious puberty is an excellent reproductive characteristic in Chinese local pig breeds; it is essential to study the regulation mechanism of precocious puberty in livestock and poultry. Chinese Hezuo pig is a typical plateau miniature pig and characterized by obviously precocious puberty. The males achieve sexual maturity at around 4 months, which is a good experimental model for studying precocious puberty traits. Testicular development directly affects the early and late sexual maturity of male animals, thus determining their reproductive performance. LncRNA has been proved to play an important regulatory role in animal reproduction [48]. Therefore, testicular development of HZ and LC boars at 30 days old and 120 days old was analyzed and compared by histology and expression characteristics of lncRNA and mRNA using RNA-seq.

The key to testicular development mainly depends on Leydig, Sertoli, and germ cells [15]. Leydig cells can stimulate testicular development and promote spermatogenesis and maturation by secreting testosterone. At the same time, Sertoli cells can provide nutrition and structural support required for the development of germ cells and secrete the androgen binding protein. Histological analysis showed that diameter of the seminiferous tube, lumen area, number of Leydig, and Sertoli and germ cells were larger in 30-day-old and 120-day-old HZ boar than those of LC boar of the same age, and sperm were observed in 120-day-old HZ boar (Figure 1). HZ boar underwent puberty development at an earlier stage than LC boar and demonstrated early sexual maturity, which is consistent with some other local pig breeds (such as Bísaro pig [49], Meishan pig [6]).

Testis development and spermatogenesis are complex biological processes, which are regulated at different developmental stages by different genes at the genetic levels, whose transcription is dynamic and stage-specific [50]. LncRNA has emerged as a major translational regulator in the reproduction process (such as gonadogenesis [51], sex hormone response [52], sex determination [53], spermatogenesis [54], and meiosis [55]) in humans and animals. In this study, a total of 10,706 lncRNAs (9282 known lncRNAs and 1424 novel lncRNAs) and 21,674 mRNAs were identified from the testis of HZ and LC boars at the age of 30 days old and 120 days old. To our knowledge, this is the first study to identify lncRNA and mRNA during testicular development in HZ boars. The large number of novel lncRNAs identified here suggests that the identification and characterization of porcine lncRNAs are still limited compared to other animals (such as mouse and rat). Since these novel lncRNAs were identified by strict criteria, they are high quality and valuable for studying the mechanisms of precocious puberty in HZ boars. In addition, lncRNA has shorter lengths, fewer exon numbers, shorter ORF lengths, and lower expression levels compared to mRNA (Figure 3A–C). These differences between lncRNA and mRNA have also been found in other mammals and may suggest some regulatory conservation in mammals [19,20,21]. We randomly selected differentially expressed eight lncRNAs and eight mRNAs to validate the accuracy of RNA-seq data using qRT-PCR. The results were consistent with the results from the RNA-seq data. These results confirmed that the identified lncRNA was of high quality and can be used to be deeply analyzed.

Based on the above results, we conferred that the transcriptomic change was caused by differences in cell types in these two breeds and two ages of testes and that lncRNA might play important roles in the regulation of sexual maturation. Previous research has looked into the functions of certain lncRNAs in testis development and spermatogenesis. For example, lncRNA *Mrhl* is negatively regulated by Wnt signaling activation through its protein partner Ddx5, which regulates the process of spermatogenesis [56]. LncRNA *Tsx* was specifically expressed in crude lineage spermatocytes, suggesting that *Tsx* plays a regulatory role in germ cell meiosis [57]. *Dmr* is a testis-specific functional lncRNA that forms trans-splicing RNA isoforms with doublesex and mab-3 related transcription factor 1 (DMRT1). This DMRT1 protein could promote spermatogonia development and prevents premature meiosis [58], indicating that lncRNA *Dmr* may be involved in the transformation of mitosis and meiosis in germ cell development.

To compare the expression profiles of lncRNA and mRNA in the testes of HZ boars and LC boars, we focused on two comparison groups, Ha vs Hb and Hb vs Lb, to screen for co-differentially expressed lncRNA and mRNA to explore some key molecules of precocious puberty traits in HZ boars. A total of 187 lncRNAs and 984 mRNAs were detected after comparison. To deeply explore the biological functions of lncRNA and mRNA, GO and KEGG analyses were performed on the target genes of these lncRNA and mRNA. The result showed that most of them were enriched in some common metabolic pathways (such as TGF-beta, PI3K-Akt, and Wnt), which play a functional role in testis development and spermatogenesis [38,39,59,60]. Through these analyses, we found some candidate lncRNAs (such as *LOC102166140*, *LOC110259451*, and *MSTRG.15011.2*) and mRNAs (such as *PDCL2*, *HSD17B4*, *SHCBP1L*, *CYP21A2*, and *SPATA3*) that could be related to the traits of precocious puberty.

Most evidence suggests that the expression of lncRNA can regulate the expression of neighboring mRNA and is highly correlated with their expression [61]. *LOC102166140* was predicted to regulate the target gene *PDCL2*. The PDCL2 protein is a member of a conserved family of small thioredoxin-like proteins. Mammalian *PDCL2* is strongly expressed in pachytene spermatocytes and early post-meiotic sperm cells in the testis, and *PDCL2* expression is restricted to male and female germ cells [62]. In our study, the expression of *LOC102166140* and *PDCL2* was significantly increased in Ha vs Hb and might positively regulate spermatogenesis by regulating the expression of *PDCL2*. *LOC110259451* was predicted to act on the target gene *HSD17B4*. The HSD17B4 protein is an enzyme protein that plays an important role in steroid metabolism, which can inactivate estradiol with high biological activity and convert it to estrone with low activity [63]. *HSD17B4* regulates the amount of intracellular estradiol secretion and maintains the stability of estrogen [64]. In this study, *LOC110259451* and *HSD17B4* genes were downregulated in mature HZ boar and might regulate sexual maturation by altering hormonal homeostasis in vivo. *SHCBP1L* is a predicted *cis*-target of the lncRNA *MSTRG.15011.2*. SHCBP1L, a conserved protein in mammals, is predominantly expressed in male germ cells and maintains spindle stability during meiosis in testis [65]. Knockout of the *SHCBP1L* gene in mice increases the number of meiosis-arrested spermatocytes [66]. High expression of *MSTRG.15011.2* and *SHCBP1L* in mature HZ boar suggested that it may promote spermatogenesis to display the precocious puberty trait.

*CYP21A2* is responsible for 21-hydroxylase activity. Mutations in *CYP21A2* can lead to 21-hydroxylase deficiency, and congenital adrenal hyperplasia (CAH) displayed early puberty [67,68]. Sunil et al. found that the E318X mutation in *CYP21A2* is associated with premature pubic hair appearance and hyperandrogenism in children [69]. The high expression of *CYP21A2* in immature HZ boars suggested that it may also be important in the precocious puberty of HZ boars. *SPATA3* is one gene of the SPATA (spermatogenesis-associated) gene family that plays a vital role in testis development and spermatogenesis [70]. *SPATA3* has been shown to be the most significantly downregulated gene in the testis of infertile patients [71]. Yang et al. found that *SPATA3* plays an important role in spermatogonia development in mice [72]. In our study, the expression of *SPATA3* was highest in mature HZ boars and significantly higher than that in LC boars of the same age. We speculated that this gene might promote spermatogenesis in cooperating pigs through apoptosis and cellular autophagy [73] to achieve precocious maturity. In addition, the constructed lncRNA–mRNA co-expression network (Figure 6) indicated that some mRNAs (such as *MSTRG.10873*, *MSTRG.11244*, and *MSTRG.10704*) were regulated by multiple lncRNA, and it was speculated that they might also play an important role in the process of precocious puberty. However, the underlying mechanisms of these potential regulatory activities need to be further investigated.

## 5. Conclusions

In summary, testis development and spermatogenesis play a vital role in the early maturation of pigs, and these functions are achieved through the regulation of different signaling pathways and related genes. We screened for related lncRNAs (such as *LOC102166140*, *LOC110259451*, and *MSTRG.15011.2*) and mRNAs (such as *PDCL2*, *HSD17B4*, *SHCBP1L*, *CYP21A2*, and *SPATA3*) that might be involved in testicular development and spermatogenesis to regulate precocious puberty traits; the useful insights could be provided for the study of the molecular mechanism of precocious puberty.

## Data Availability

The data presented in this study are available in the Gene Expression Omnibus (GEO) of the National Center for Biotechnology Information (NCBI) [accession number: GSE171756].

## Supplementary Materials

The following are available online at https://www.mdpi.com/article/10.3390/ani11082263/s1, Figure S1: the distribution of mapped reads on gene (5′–3′), Figure S2: the ratio of reads mapped to different regions of the genome, Figure S3: schematic diagram of the pipeline for identifying lncRNA, Figure S4: PCA plot of lncRNAs (A) and mRNAs (B) expressed in each sample, Figure S5: schematic diagram of the pipeline for bioinformatics analysis, Table S1: primer sequences used in this study, Table S2: diameter of seminiferous tubules in testes of Hezuo boar and Landrace boar of different ages, number of spermatogenic cells and Sertoli cells, Table S3: summary of raw reads after quality control and mapping to the reference genome, Table S4: GO annotation of differentially expressed mRNA, Table S5: KEGG pathway enrichment of differentially expressed mRNA, Table S6: prediction of cis-target genes for lncRNA, Table S7: GO annotation of cis-target genes of lncRNA, Table S8: KEGG pathway enrichment of cis-target genes of lncRNA, Table S9: prediction of trans-target genes for lncRNA, Table S10: GO annotation of trans-target genes of lncRNA, Table S11: KEGG pathway enrichment of trans-target genes of lncRNA, Table S12: list of candidate molecules screened for precocious puberty.
