# Supplementary material for: Identification and Characterization of lncRNA and mRNA in Testes of Landrace and Hezuo Boars
Source: Animals (Basel). 2021 Jul 30;11(8):2263. doi: 10.3390/ani11082263 (PMC8388364; doi:10.3390/ani11082263)
Supplement: Supplementary file 1 [file animals-11-02263-s001.zip › Table S2.pdf]

Table S2. Diameter of seminiferous tubules in testes of Hezuo boar and Landrace boar of different ages, number of spermatogenic cells and Sertoli cells (mean ± SEM).

Parameters	Hezuo boar (HZ)		Landrace boar (LC)	
	30-day-old	120-day-old	30-day-old	120-day-old
Diameter of Spermatogenic Tubules (μm)	80.85±4.83[b]	176.37±17.31[a]	56.53±2.55[b]	152.11±2.88[a]
Number of Spermatogenic Cells	21.78±1.09[c]	149.78±3.61[a]	15.00±1.58[c]	98.89±3.95[b]
Number of Sertoli Cells	1.78±0.44[b]	8.44±0.91[a]	2.11±0.22[b]	4.75±0.91[a]

* In each row, values with different superscripts are significantly different ($P < 0.05$).
