# Supplementary figures and images for: Identification and Characterization of lncRNA and mRNA in Testes of Landrace and Hezuo Boars
Source: Animals (Basel). 2021 Jul 30;11(8):2263. doi: 10.3390/ani11082263 (PMC8388364; doi:10.3390/ani11082263)

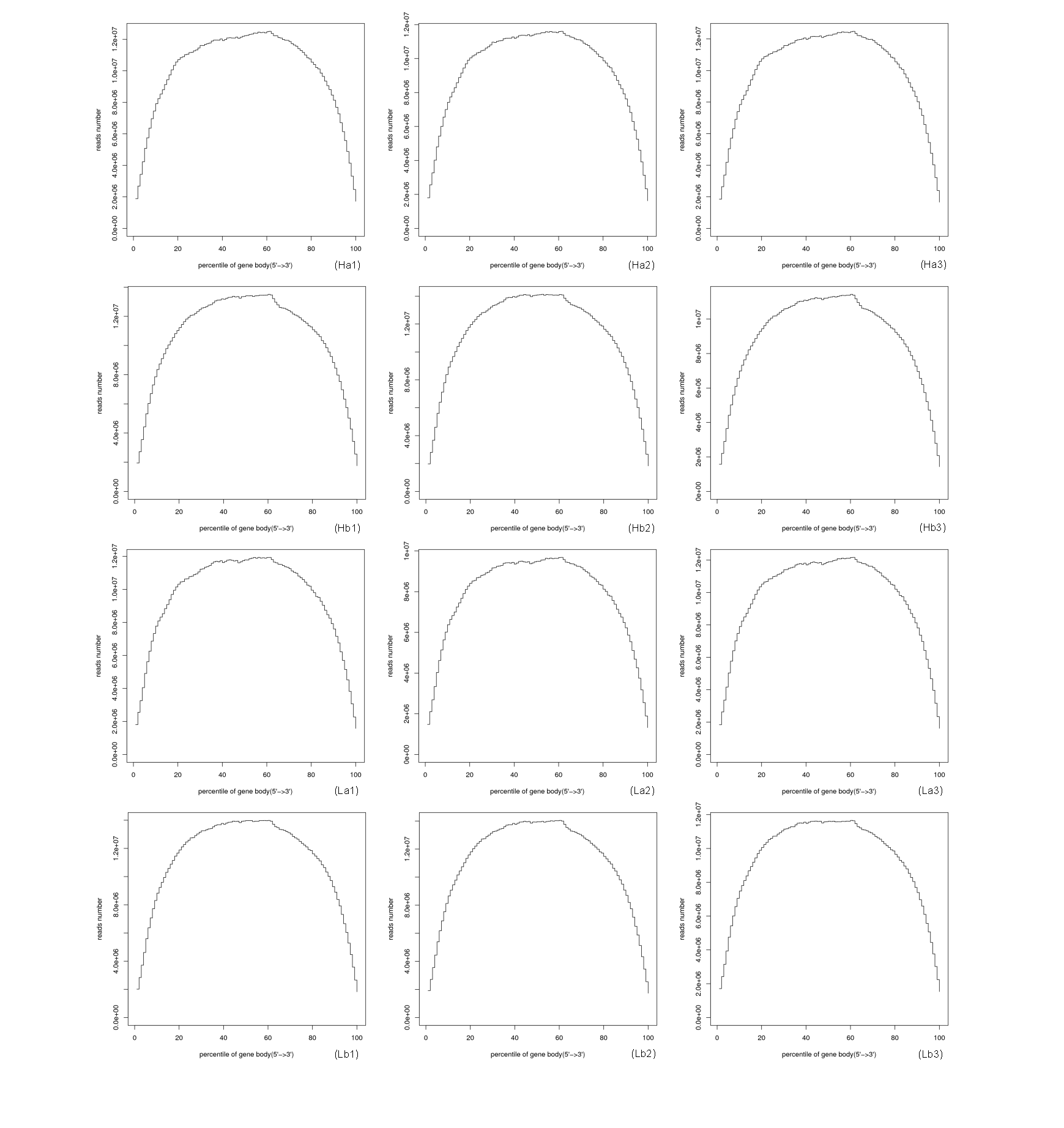

Supplement: Supplementary file 1 [file animals-11-02263-s001.zip › Figure S1.tif]

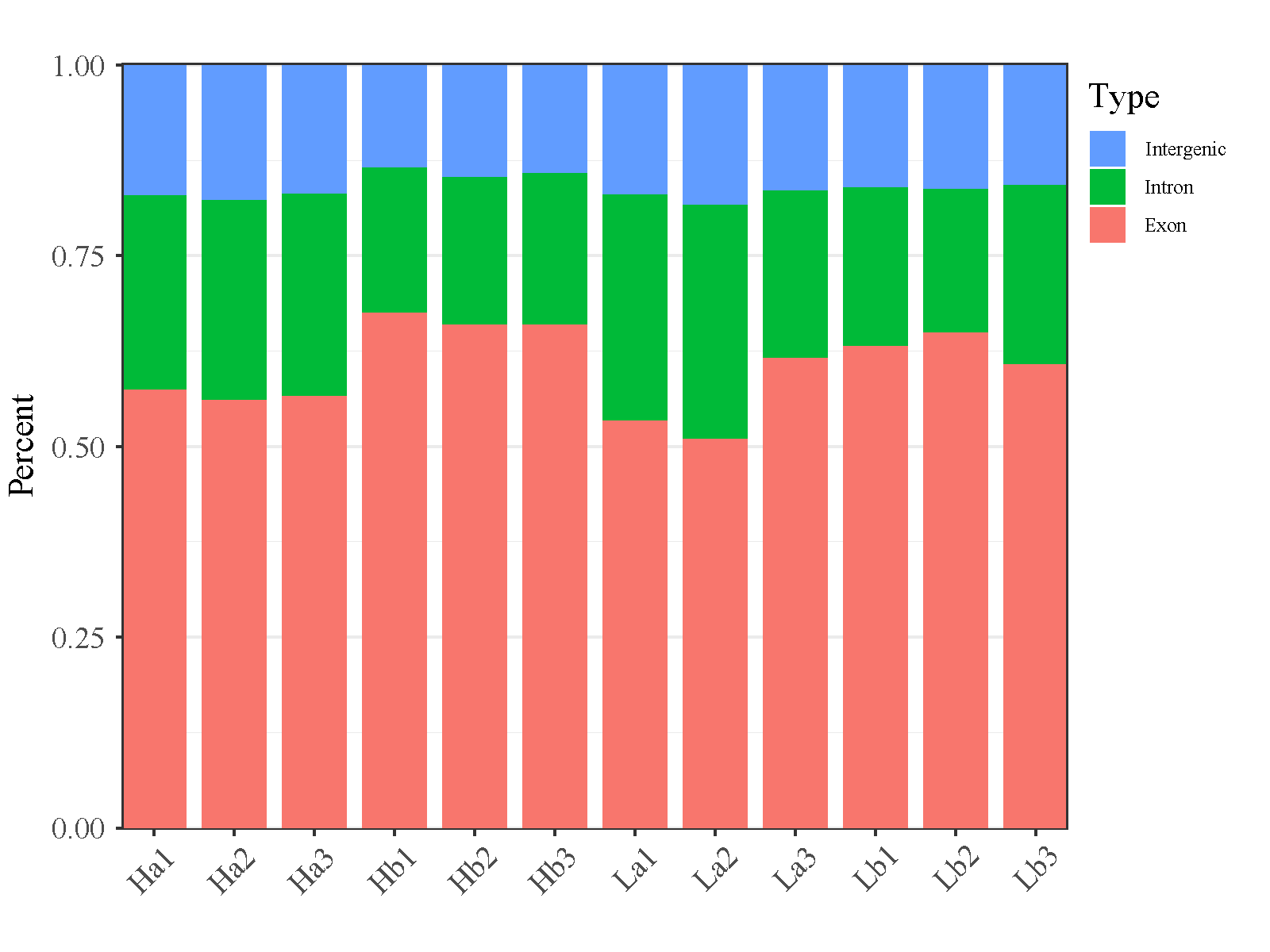

Supplement: Supplementary file 1 [file animals-11-02263-s001.zip › Figure S2.tif]

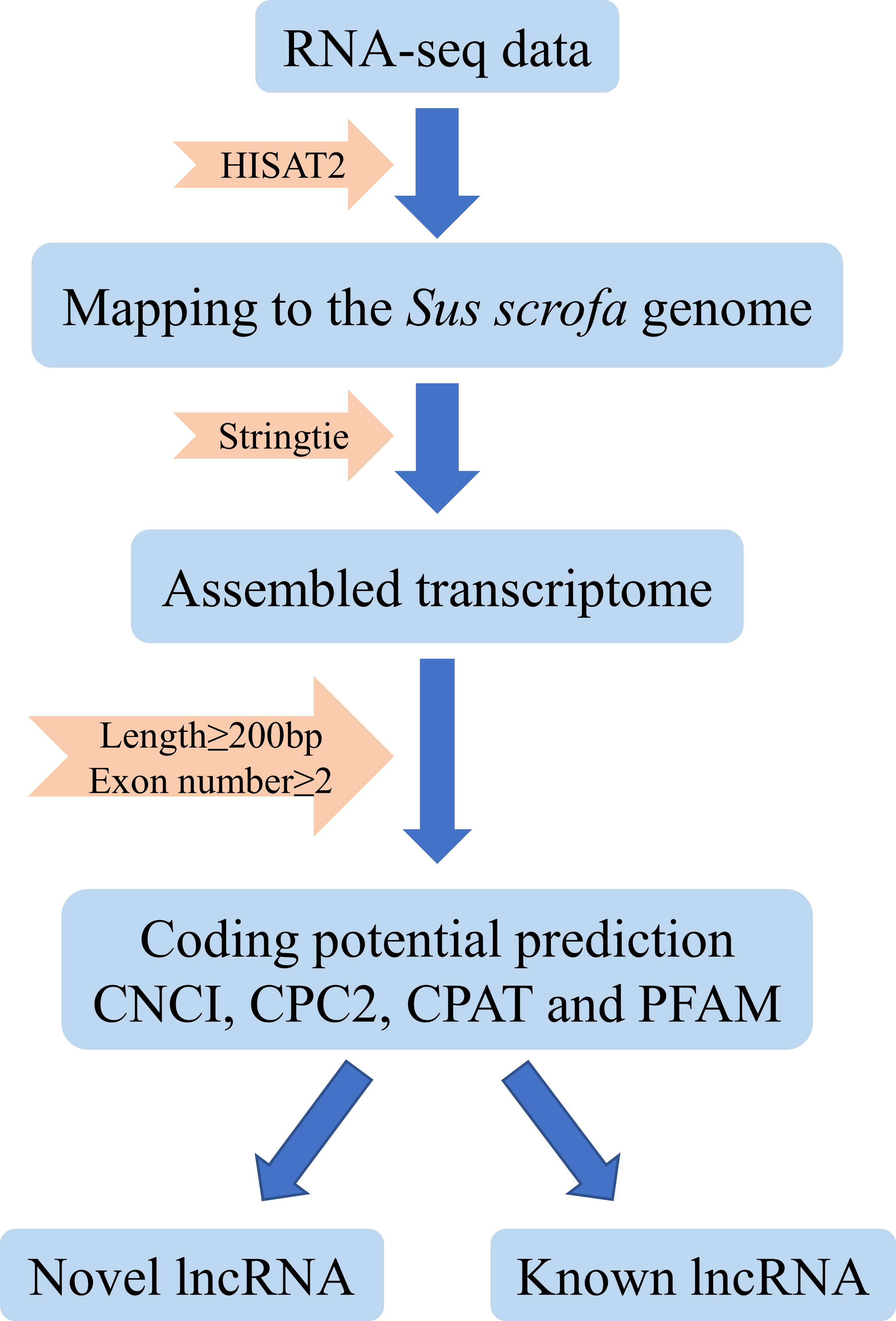

Supplement: Supplementary file 1 [file animals-11-02263-s001.zip › Figure S3.tif]

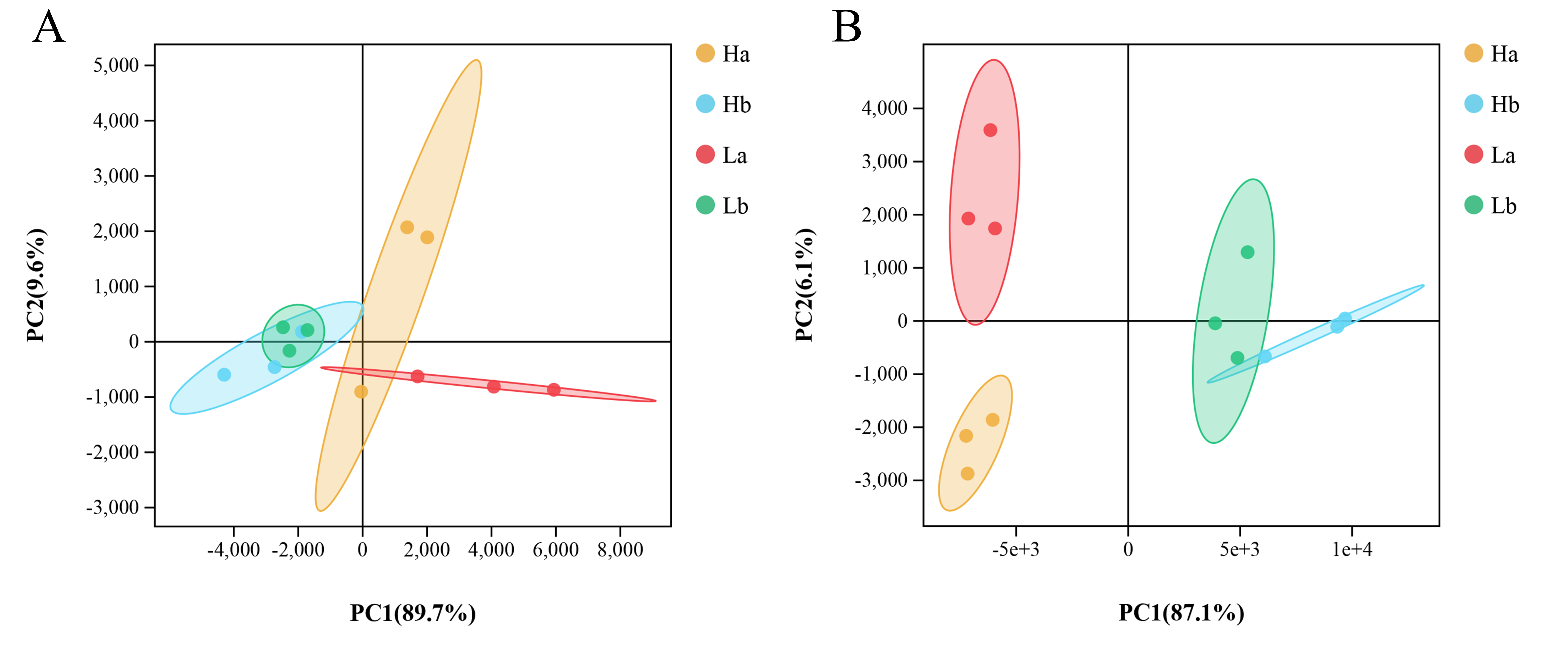

Supplement: Supplementary file 1 [file animals-11-02263-s001.zip › Figure S4.tif]

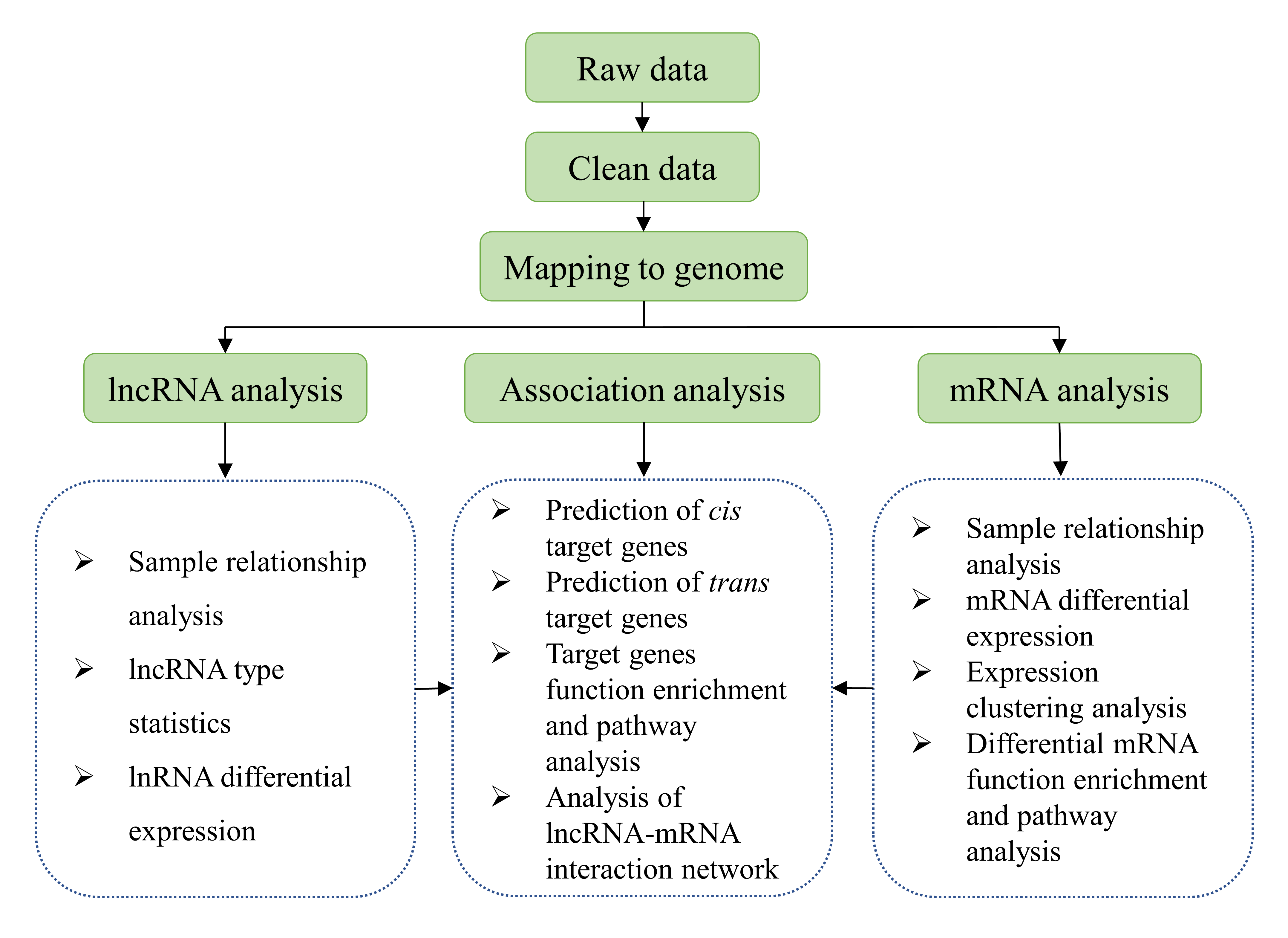

Supplement: Supplementary file 1 [file animals-11-02263-s001.zip › Figure S5.tif]
